# What do European guidelines say about genetic testing for people with mental disorders? A scoping review

**DOI:** 10.1192/j.eurpsy.2025.1528

**Published:** 2025-08-26

**Authors:** I. Arican, J. Zinkstok, M. van der Horst, N. Bass

**Affiliations:** 1 Division of Psychiatry, University College London, London, United Kingdom; 2 Department of Psychiatry, University Medical Center Utrecht, Utrecht, Netherlands

## Abstract

**Introduction:**

Technological advancements have identified numerous genetic variations linked to mental disorders, providing potential explanations and, in some cases, enabling targeted treatments. However, clinical genetic testing remains underutilised in psychiatric care, potentially due to inconsistent clinical guidelines across Europe.

**Objectives:**

This scoping review aims to compile, summarise and evaluate European clinical practice guidelines (CPGs) on genetic testing in mental disorders, identifying gaps and variations in recommendations to inform current practice and future guideline development.

**Methods:**

A scoping review was conducted across scientific databases (PubMed/MEDLINE and Ovid) and grey literature sources (Image 1. Flow diagram). Inclusion criteria centred on European CPGs published in English from 2010 onward with specific recommendations on genetic testing in mental disorders. Quality assessment was performed using the International Centre for Allied Health Evidence (iCAHE) checklist. Data extraction focused on guideline characteristics, target populations, and genetic test recommendations.

**Results:**

Sixteen CPGs met the inclusion criteria, displaying considerable heterogeneity in quality and content, and covering a limited range of mental disorders. Six guidelines addressed neurodevelopmental disorders. Most recommended genetic testing in Autism Spectrum Disorder (ASD) when indicators such as intellectual disability (ID) or dysmorphic features were present; however, one guideline recommended routine testing. Only one guideline included recommendations for genetic testing in ID; routine access to Fragile X testing, chromosomal microarray, and whole genome sequencing was recommended as standard care.

Eleven guidelines provided recommendations on genetic testing in neurodegenerative disorders. In dementia, consensus on routine testing was generally limited to young-onset cases or those with distinct genetic profiles. APOE genotyping was generally discouraged. Guidelines for diagnostic testing for Huntington’s Disease (HD) were consistent. Access to predictive testing with appropriate genetic counselling for at-risk adults was also recommended.

**Image:**

**Figure:**
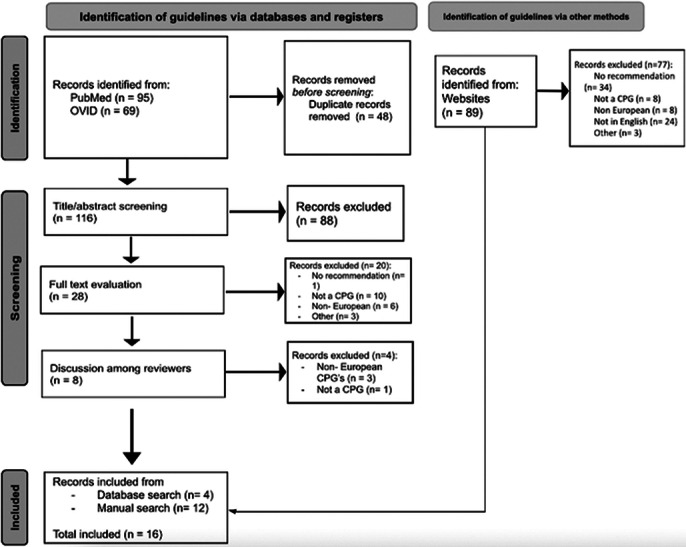

**Conclusions:**

Based on our findings and the wider literature, we recommend considering genetic testing for: 1) all patients with ID, 2) patients with ASD exhibiting features suggestive of a genetic cause, such as ID and dysmorphic traits, and 3) patients with dementia with a young age of onset or a family history indicative of a Mendelian disorder. For HD, testing should be informed by phenotypic features and family history. Establishing harmonised, evidence-based guidelines is essential to integrate testing effectively. Key considerations include clinical utility, patient autonomy, and access to genetic counselling to ensure informed and supportive care.

**Disclosure of Interest:**

None Declared

